# Recent Advancement of Electromagnetic Interference (EMI) Shielding of Two Dimensional (2D) MXene and Graphene Aerogel Composites

**DOI:** 10.3390/nano10040702

**Published:** 2020-04-08

**Authors:** Kanthasamy Raagulan, Bo Mi Kim, Kyu Yun Chai

**Affiliations:** 1 Division of Bio−Nanochemistry, College of Natural Sciences, Wonkwang University, Iksan 570-749, Korea; raagulan@live.com; 2 Department of Chemical Engineering, Wonkwang University, Iksan 570-749, Korea

**Keywords:** MXene, Graphene, EMI shielding, aerogel, composites

## Abstract

The two Dimensional (2D) materials such as MXene and graphene, are most promising materials, as they have attractive properties and attract numerous application areas like sensors, supper capacitors, displays, wearable devices, batteries, and Electromagnetic Interference (EMI) shielding. The proliferation of wireless communication and smart electronic systems urge the world to develop light weight, flexible, cost effective EMI shielding materials. The MXene and graphene mixed with polymers, nanoparticles, carbon nanomaterial, nanowires, and ions are used to create materials with different structural features under different fabrication techniques. The aerogel based hybrid composites of MXene and graphene are critically reviewed and correlate with structure, role of size, thickness, effect of processing technique, and interfacial interaction in shielding efficiency. Further, freeze drying, pyrolysis and hydrothermal treatment is a powerful tool to create excellent EMI shielding aerogels. We present here a review of MXene and graphene with various polymers and nanomaterials and their EMI shielding performances. This will help to develop a more suitable composite for modern electronic systems.

## 1. Introduction

The proliferation of smart electronic devices and wireless communication in the artificial intelligent age are a source of electromagnetic pollution (EMP) which is a serious universal problem. The EMP creates complexities in the natural electromagnetic environment, and is unwanted radiation which not only disturbs the general function of surrounding electronic systems but also threatens the well−being of humans [1,2,3,4,5,6,7,8]. This disturbing phenomenon is called electromagnetic interference (EMI) and it causes data theft, malfunction of the electronic devices, degradation of basic function of electronic devices, and vulnerability of the personal security in electronic components, while grounding problems in humans such as mutation, insomnia, headache, leukemia, damage the organs, thermal injuries, and cancer [1,2,3,4,5]. In addition, expansion of digital networks and sensitive remote-controlled systems demanding high quality densely built electronic control systems, creates un-compatible environments (UCE) or electromagnetic noise (EMN) or EMI and the current of electrodynamic and basic field are essential sources of EMI. At low frequency where electric and magnetic fields act independently, whereas high frequency waves are propagating electromagnetic radiation (EMR) which causes EMI [9]. The source of the EMI are radio and TV transmission, radar, aviation, electromagnetic missiles, warfront, Bluetooth, wireless network (WLAN), remote controls in which EMI occur by radiative coupling. The radiative coupling is due to the disturbance in all conductive parts by propagating high frequency radiation. In addition, the other types of coupling such as inductive, impedance and capacitive which are due to the current, voltage, and resistance in electric circuits [9]. 

In 2020, the world utilizes electromagnetic radiation as a jamming tool or weapon in battlefields or to destroy electronic components of the ships, radars, and flights, and destroy security of the countries. Hence, electromagnetic interference shielding is the inevitable choice around the globe [9,10,11,12,13,14,15]. So far, scientists have studied EMI shielding in various frequency range by using numerous materials which include zero−dimension (0D), one−dimension (1D), two−dimension (2D), and three−dimension (3D) materials. The combination of these materials gives rise to different structural feature influences with excellent EMI shielding behavior. The MXene, MAX phase, quantum dots (QDs) metal nanoparticle, oxide nanoparticle, carbon black, graphene (GN), graphite, single wall carbon nanotube (SWCNT), multiwall carbon nanotube (MWCNT), magnetic nanoparticle, metal oxides, core shell Nano materials, metal plates, organic substances, nonconductive polymers (NCP), conductive polymers (CP), and craft polymers are being utilized for EMI shielding application. Graft and nonconductive polymers are being used as a matrix and used to connect the (0, 1, 2, and 3)−dimensional materials (Table 1) [10,11,12,13,14,15,16,17,18,19]. CPs which act as fillers and help to create different structural feature meanwhile electric conductivity (EC), thermal conductivity (TC), tensile strength, and EMI shielding of the composites also improved substantially. In addition, the different organic substances and nanomaterials are being used to improve adhesivity, dispersity, porosity, and other physiochemical properties of the composites [1,20,21].

**Figure 1:**
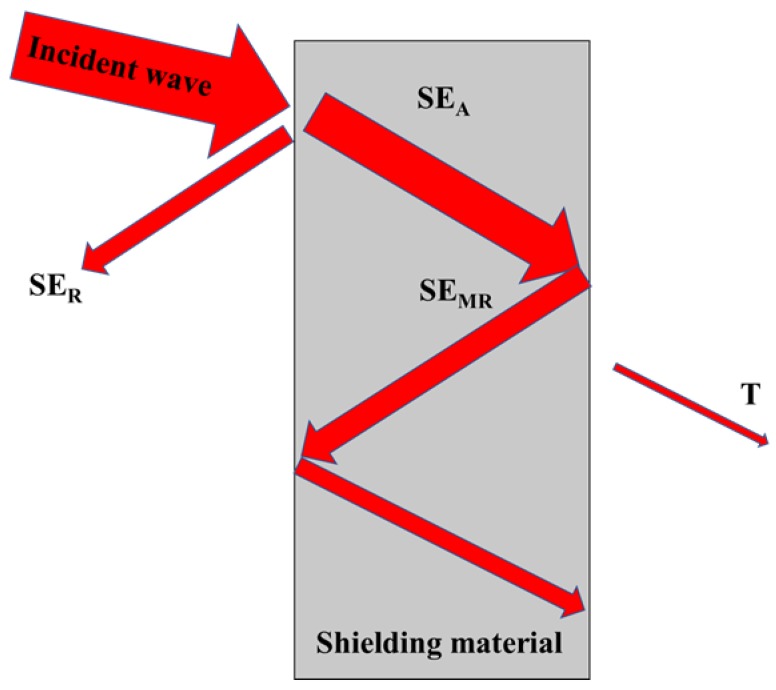
Transformation of the electromagnetic radiation (EMR) at the shielding material.

EMI shielding can be achieved by absorption (SE_A_), reflection (SE_R_), and multiple reflection (SE_MR_). SE_R_ occurs on the surface whereas SE_A_ and SE_MR_ are happening within the shielding material. The wave propagating on the other side of the shielding materials is called the transmittance (T), the magnitude of which is lower than that of incident wave (I) and value of SE_A_, SE_R_, and SE_MR_ is smaller than that of I (Figure 1). The electrical conductivity of the composite responsible for the reflection while porous in structure, electric and magnetic bipolarity of the composite and thickness are responsible for the absorption and multiple reflection occurs due to the porous nature and multiple layer structure [1,13,15,16,17,18]. In addition, the thickness of the shielding material is higher than that of the skin depth leading to multiple reflection and finally roots the absorption and is a negligible component compared to the absorption and reflection. Though, one of the factors domineers over others which is dependent on the types of material being used [19,20,21]. Most of the studies show that the absorption is higher than that of the reflection and a recent study disclosed that the reflection can be eliminated from the basic mechanism, and absorption only determined the total EMI shielding. This phenomenon can be achieved by creating different internal structure with higher electric conductivity. The pure material showed higher EMI shielding, but, when a foreign material is introduced into the pure material the EMI shielding is considerably reduced by those foreign polymers. Even though, without polymers or binders, it is difficult to use EMI shielding applications in different electronic devices [1,13,15,17,18,19,20].

We focused on a different synthetic route of graphene (GN), MXene, and composites. GN and MXene structural features and synthesis are discussed in detail. In addition, different composite preparation methods are listed, and its effect is discussed in detail with proper comparison in the discussion. In general, aerogel or foam based composite are most attractive to scientists and have excellent EMI shielding with excellent absorption ability. 

## 2. Results and Discussion

### 2.1. Synthesis of Graphene (GN) and Structural Features

Top−down and bottom–up approaches are being used to synthesize graphene. The top−down approach where graphite is used as a precursor and mechanical exfoliation, graphite intercalation, nanotube slicing, pyrolysis method, reduction of the graphene oxide (GNO), electrochemical exfoliation, sonication, radiation based method, and ball−milling are being used to produce GN while bottom–up follows the strategy that those are grown from metal−carbon melt, epitaxial growth on silicon carbide (SiC), dry ice method, and deposition (Table 2). Multilayer highly ordered pyrolytic graphite (HOPG) is scraped off to produce GN by mechanical exfoliation for which scotch tape, ultrasonic oscillations, and hot press techniques are being used. In the mechanical exfoliation process two types of normal force used to peel of the graphite and shear force utilize ball milling process [9,22,23,24,25]. The liquid phase exfoliation (LPE) method includes three basic processes, those are dispersion of graphite in suitable solvent, exfoliation, and purifying where van der Waals forces break down by solvents and ultrasound. The arc discharge method is utilized to synthesize the allotropes of carbon such as graphene, CNT, and fullerene. In which, the carbon precursor is used as the anode and the graphite rod as the cathode, where applied electrical current creates the plasma at higher temperature (3727–5727 °C) and finally form graphene. Various intercalants are being utilized by the intercalation technique. By carbon nanotube (CNT) slicing/unzipping micron size GN are produced for which plasma or chemical etching, intercalation and exfoliation, metal catalyst cutting, abrade on the glass surface, and the CNT tube opens into a lay-flat single layer graphene ribbon (GNR). The pyrolysis is the type of the solvothermal technique where equal molar sodium and ethanol are used to separate the graphite layers. The thermal annealing technique is where amorphous carbon is converted into single layer graphene on the nickel and cobalt surface with the aid of temperature. The reduction of the graphene oxide (GO) can be performed by using chemicals, biomass, radiation, bacteria, electrochemical methods, and heat treatment. Electrochemical exfoliation is practiced in an acidic environment by applying voltage differences between the anode (graphite) and cathode (platinum) while solvent-based high energy is used in the sonication technique. The ball milling utilized solvent or chemical assistance and magnetic assistance technique, although higher quality GN are being produced by using radiation techniques. Laser and electron beams are used in radiation techniques. The deposition method utilizes the Chemical Vapor Deposition (CVD) techniques where solid, liquid, and gaseous carbon precursors are being used, filtration with reduction, spin coating, and spray coating process for GN synthesis. The CVD produces graphene with low defects and during the process the precursors are atomized, and the graphene is formed on the metal catalyst (Cu and Ni). The epitaxial growth is performed on a silicon carbide wafer where stacks of graphene are formed. The epitaxial growth is the exothermic process during which the sublimated (1200–1600 °C) silicon leaves excessive sp^2^ hybridized carbon network formed graphene. In addition, burning the dry ice by using magnesium followed by acid treatment produces GN (Table 2) [9,23,24,25,26,27].

**Figure 2:**
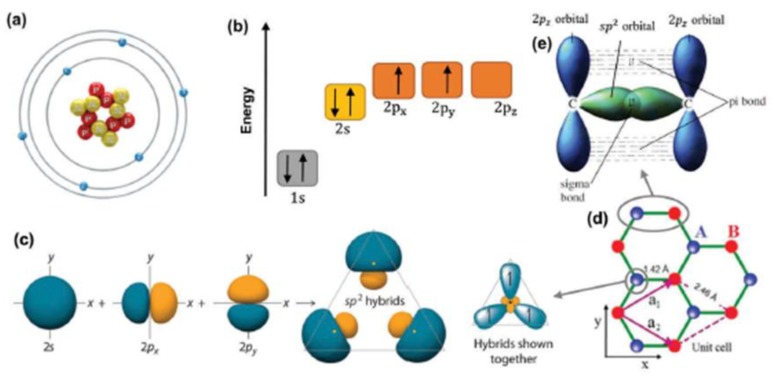
(**a**). Atomic structure of a carbon atom. (**b**) Energy levels of outer electrons in carbon atoms. (**c**) The formation of sp^2^ hybrids. (**d**) The crystal lattice of graphene, where A and B are carbon atoms belonging to different sub−lattices, a1 and a2 are unit−cell vectors. (**e**) Sigma bond and pi bond formed by sp^2^ hybridization [29] Copyright Science and technology of advanced materials, 2018.

**Figure 3:**
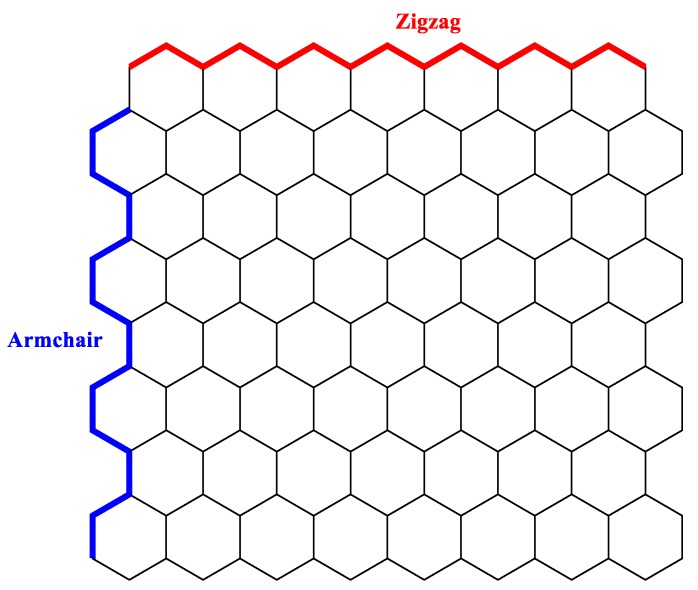
Zigzag–edged and armchair–edged of graphene (GNs).

**Figure 4:**
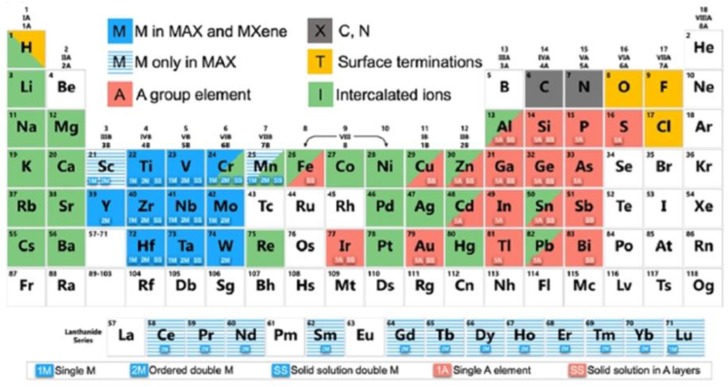
Elements used to create MAX phases, MXenes, and their intercalated ions. The A elements are denoted by a red background and are used to synthesize MAX phases that can possibly be utilized to make MXenes. The elements denoted by a green background, have been intercalated into MXenes (to date) and the symbols are at the bottom, 1M and 1A designate the formation of a single (pure) transition metal and A element MAX phase (and MXene). Solid solutions are indicated by an SS in transition metal atomic planes (blue) or A element planes (red); and 2M indicates the formation of an ordered double-transition metal MAX phase or MXene (in-plane or out-of-plane). The MAX phase elements denoted by blue striped background have not yet been used to synthesis MXene (Figure 5) [32] Copyright American Chemical Society, 2019.

The graphene (GN) is a 2D single layer crystalline material with a honeycomb (HC) structure, comprised of sp^2^ hybridized carbon atoms. In early 1947, Wallance et al. reported the 2D graphite with zero activation energy by using “tight binding” approximation which was later experimentally observed in 2004 [28,29,30,31]. Due the excellent properties of GN, it is an excellent candidate for use in modern electronics. There are two major types of GN, those are zigzag and armchair [28]. Figure 2 exhibits the electrons in carbon atoms (Figure 2a), energy comparison at ground state electronic configuration (Figure 2b), shape of the orbitals and hybridized orbitals (Figure 2c), crystal lattice and unit cells (Figure 2d), and bond formation (*σ* and *π*) (Figure 2e). The carbon is a group IV element on the periodic table, with 1s^2^, 2s^2^ sp^2^ ground state electronic configuration where all 2p orbitals are regenerated and 2p_z_ is an empty orbital while each 2p_x_ and 2p_y_ holding one electron, respectively. The empty 2p_z_ orbital plays a major role in creating out of the plane *π* bond and sp^2^ hybridized carbon form in−plane *σ* bond which extends to hexagonal web of carboned leads to formation of mono layer graphene. The average inter atomic distance of GN is 1.42 Å, thus the graphene co−valent bond is stronger than that of C−C bond of alkanes [29]. The monolayer graphene possesses 130.5 GPa of intrinsic tensile strength and 1 TPa of Young’s modulus. In addition, the monolayer GN has no band gap which is due to the free moving *π* electrons which offer weak van der Waals forces between the graphene layers [29]. Due to the weak Van der Waals forces the graphene can be synthesized from bulk graphite (Figure 3 and Figure 4) [28,29,30].

### 2.2. MXene Synthesis and Structural Features.

**Figure 5:**
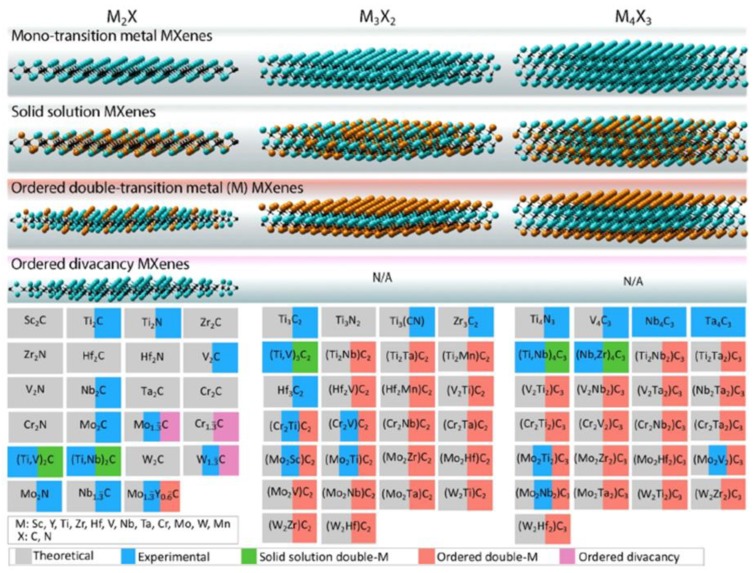
MXenes synthesized up to date. The top row illustrates structures of (top–down) mono−M MXenes, double-M solid solutions (SS) (marked in green), ordered double-M MXenes (marked in red), and ordered divacancy structure (only for the M_2_C MXenes), respectively. This table shows the MXene reported both experimentally (blue) and theoretically (gray) so far [32] Copyright American Chemical Society, 2019.

**Figure 6:**
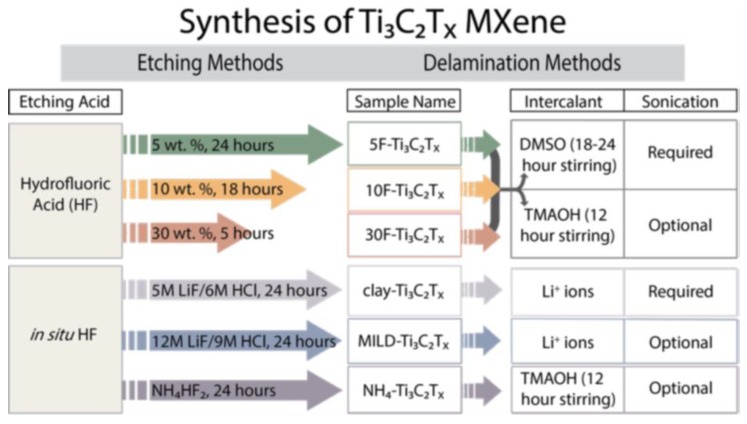
The general MXene synthetic route with both the HF and in−situ HF etching process and the delamination process [33] Copyright American Chemical Society, 2017.

The MXene is a fast-growing two−dimensional (2D) material, derived from its corresponding 3D MAX phase by an etching process. The general formula of MXene is M_n+1_X_n_T_x_ where M is an early transition element, n = 1–3, X is a carbon or nitrogen and T_x_ is a surface functional groups −F, −OH, =O, and Cl, which are directly attached to the M. The M_n+1_AX_n_ is used to denote the MAX phase where generally is group A 13/14 element, but, several other elements also utilize for A layer (Figure 4 and Table 1). The different combination of elements has been used for both MAX and MXene synthesis shown below in Figure 5 and Figure 6. The number of layers in MXene is determined by n where the n+1 layer of MXene is formed which is true for the MAX phase as well (Figure 5) [31,32,33].

**Figure 7:**
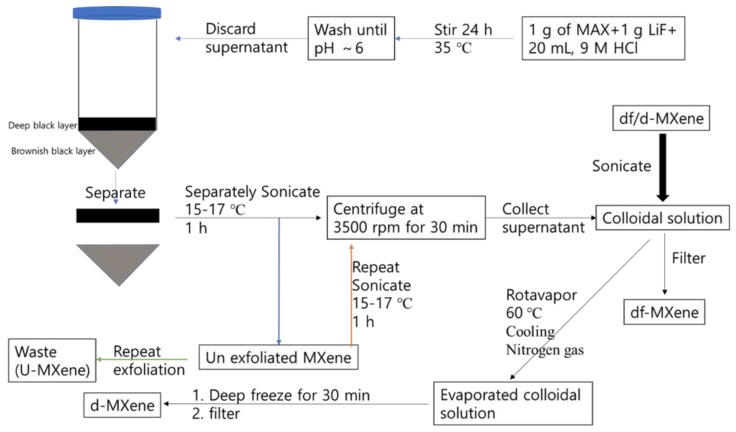
Flow chart of the synthetic approach of the exfoliated MXene synthesis [31] Copyright Royal Society of Chemistry, 2020.

During the development of MXene scientists practiced various strategies to produce good quality MXene. During the etching process, the A layer of the MAX phase is eradicated and surface functionalities are introduced. Fluoride based etchants are being used for the etching process, those are HF, NH_4_HF_2_, LiF/HCl, and FeF_3_/HCl. The HF based etching process causes risk compared to the in situ etching process where fluoride salt and acid are used. According to HF protocol, 1 g of Ti_3_AlC_2_ is mixed with 20 mL of the HF etchant, and the concentration of HF varies based on its requirement. The 10% of HF is enough to remove Al with accordion−like morphology while 5% of HF is not enough to get accordion-like morphology, but is good enough to remove Al. The in−situ process based on the salt/acid etching process where HF is formed during the reaction is called minimally intensive layer delamination (MILD). The LiF/HCl reaction is being widely used as it produces less defective MXene compared to other studies. Recently, most of the scientists around the globe use 9M HCl/LiF as a standard. Exfoliation is a process where single the MXene layer is separated by various process such as intercalation and sonication. The sonication is the physical method where MXene is ultra-sonicated in water while intercalation used organic molecules followed by sonication. Dimethyl sulfoxide (DMSO), tetrabutylammonium hydroxide (TBAOH), tetramethylammonium hydroxide (TMAOH), and urea are widely used in the HF based etching process, whereas the MILD method does not need the intercalation process utilized in sonication at about 15 °C under an inert environment and lithium ion induced exfoliation process (Figure 6). Raagulan et al. reported a method for the mass production of exfoliated MXene and its by-products. In which, the evaporation technique is used to concentrate the colloidal solution and filtration process is explored to separate solvent and delaminated MXene (Figure 7) [31,32,33]. 

### 2.3. EMI Shielding Theory and Mechanisms

The EMI shielding can be defined as how well a material quantitatively weakens the energy of the propagating electromagnetic radiation (EMR) in a certain frequency range (L, S, C, X, K, Ku, etc. bands). The EMR with specific power (P_I_) hits the surface of the shielding materials undergoing different transformations such as reflection (P_R_), absorption (P_A_), and transmittance (P_T_). The reflection occurs on the surface while absorption happens within the materials and remainder comes out as transmittance EMR [34]. 

The absorption (*A*) is depend on the type of material used. The *A* and absorption coefficient (*A_e_*) can be expressed as follows, where reflection (*R*) and transmittance (*T*) are correlated (Equations (1) and (2)) [35].

(1) A=1−R

(2) $$A_{e}=\left[\frac{1-R-T}{1-R}\right]$$

The attenuation of EMR is expressed by shielding effectiveness (*SE*) and the corresponding unit is given in dB. According to the EMI shielding theory, the *SE* could be defined as the logarithmic ratio between *P_i_* and power of transmittance (*P_t_*) and can also be defined by using electric intensity (*E*), magnetic intensity (*H*), wavelength (*λ*), and slot length (*l*) of the EMR [36]. The total shielding effectiveness (*SE_T_*) is expressed by using the following equations where *i* and *t* are denoted as incident and transmittance waves, respectively (Equation (3)) [37].

(3) $$SE_{T}=10\log\left(\frac{P_{i}}{P_{t}}\right)=20\log\left(\frac{E_{i}}{E_{t}}\right)=20\log\left(\frac{H_{i}}{H_{t}}\right)=20\log\left(\frac{\lambda }{2l}\right)$$

Further, *SE_T_* can be calculated by adding reflection (*SE_R_*), absorption (*SE_A_*), and multiple reflection (*SE_MR_*) (Equation (4)).

(4) $$SE_{T}=SE_{R}+SE_{A}+SE_{MR}$$

If the *SE_T_* > 15 dB, the *SE_M_* is neglected and equation can be written as follow (Equation (5)),

(5) $$SE_{T}=SE_{R}+SE_{A}$$

Moreover, the *T* and *R* can be expressed by using electric intensity (*E*) and scattering parameters in which *t* is the transmittance wave, *i* is the incident wave and *r* is the reflection wave (Equations (6) and (7)).

(6) $$T={\left|\frac{E_{t}}{E_{i}}\right|}^{2}={\left|S_{12}\right|}^{2}={\left|S_{21}\right|}^{2}$$

(7) $$R={\left|\frac{E_{r}}{E_{i}}\right|}^{2}={\left|S_{11}\right|}^{2}={\left|S_{22}\right|}^{2}$$

Further, the *SE_T_*, *SE_R_*, and *SE_MR_* can be expressed in terms of scattering parameters, wave impedance of air (*Z_o_*), wave impedance of the material (*Z_m_*), propagation constant (*β*), relative magnetic permeability (*μ_r_*), thickness of the shielding materials (*t*), and imaginary unit (*j*) (Equations (8)–(10)).

(8) $$SE_{T}=10\log\left(T\right)=SE_{R}+SE_{A}=10\log\left(\frac{1}{1-{\left|S_{12}\right|}^{2}}\right)=10\log\left(\frac{1}{{\left|S_{21}\right|}^{2}}\right)$$

(9) $$SE_{R}=10\log\left(1-R\right)=\left(\frac{1}{1-{\left|S_{11}\right|}^{2}}\right)=20\log\left|\frac{{\left(Z_{o}+Z_{m}\right)}^{2}}{4Z_{o}Z_{m}}\right|≅20\log\left|\frac{Z_{o}}{4Z_{m}}\right|$$

(10) $$SE_{M}=20\log\left(\frac{1}{4}\sqrt{\frac{\sigma }{\omega \mu_{r}\varepsilon_{o}}}\right)=20\log\left|1-{\left(\frac{Z_{o}-Z_{m}}{Z_{o}+Z_{m}}\right)}^{2}e^{-2t/\delta }e^{-2j\beta t}\right|=20\log\left|1-e^{-2t/\delta }\right|$$

In addition, the *SE_A_*, *SE_R_*, and *SE_MR_* can differently be described by using parameters of the shielding materials such as t, skin depth (▯), *μ_r_*, refractive index (*n*), relative conductivity (*σ_r_*), and imaginary part of wave vector (*ik*) (Equations (11)–(13)).

(11) $$SE_{A}=\frac{8.7t}{▯}=131.4d\sqrt{f\mu_{r}\sigma_{r}}=K\left(\frac{t}{\delta }\right)=10\log\left[\frac{T}{\left(1-R\right)}\right]=10\log\left(1-A_{e}\right)=20\log e^{t/\delta }=20lm\left(k\right)d\;\log\;e$$

(12) $$SE_{R}=108+\log\left(\frac{\sigma }{f\mu }\right)=39.5+10\log\left(\frac{\sigma }{2\pi f\mu }\right)=20\log\left|\frac{1+n^{2}}{4n}\right|$$

(13) $$SE_{M}=20\log\left|1-10^{\frac{SE_{A}}{10}}\right|=168+10\log\left(\frac{\sigma_{r}}{\mu f}\right)=20\log\left|\frac{1-\left(1-n^{2\;}\right)}{{\left(1+n\right)}^{2}}\exp\left(2ikd\right)\right|$$

Skin depth is inversely proportional to square root of *πfσμ* of the composition where *f* is frequency of EMR, *μ* is magnetic permeability, and *σ* is electric conductivity (Equation (14)) [36].

(14) $$▯=\frac{1}{\sqrt{\pi f\sigma \mu }}$$

When the electromagnetic radiation propagates, it undergoes the changes from near field to far field which is depend on the distance. The *r* < *λ*/2*π* is considered as near field and *r* > *λ*/2*π* is denoted as far field. Thus, most of the EMRs are far field and are regarded as planar waves. The impedance of the wave (intrinsic impedance) *Z* can be articulated that the amplitude ratio between electric fielding (*E*) and magnetic field (*H*) waves, which are perpendicular to each other (*E*┴*H*). Furthermore, the *Z* is influenced by *σ*, *μ*, angular frequency (*ω* = 2*πf*), *j*, and electric permeability (*ε*). *Z* of air is symbolized as *Z_o_*, and has a value of 377 Ω and at this stage *j* and *ω* is considered as one and *σ* is zero (Equations (15)–(17)).

(15) $$Z=\frac{\left|E\right|}{\left|H\right|}$$

(16) $$Z=\sqrt{\frac{j\omega \mu }{\sigma -j\omega \varepsilon }}$$

(17) $$Z_{o}=\sqrt{\frac{\mu_{o}}{\varepsilon_{o}}}$$

The EMI shielding of the composites are complicated and the physiochemical properties of constitutional composition of the composites which are significantly different from the homogeneous shielding materials. The most imperative parameter for the theoretical calculation of the EMI shielding is the effective relative permittivity *ε_eff_* of the composite that can be calculated by using the Maxwell Garnett formula. The *ε_eff_* is determined by the relative permittivity of the matrix (*ε_e_*), relative permittivity of the fillers (*ε_i_*), and *f* is the volume fraction of the filler. The *ε_i_* is calculated by using the imaginary part of the complex relative permittivity (*ε*′ and *ε*″), imaginary unit (*j*), *σ*, *ω*, and *ε_o_* (Equations (18) and (19)).

(18) $$\varepsilon_{eff}=\varepsilon_{e}+3f\varepsilon_{e}\frac{\varepsilon_{i}-\varepsilon_{e}}{\varepsilon_{i}+2\varepsilon_{e}-f\left(\varepsilon_{i}-\varepsilon_{e}\right)}$$

(19) $$\varepsilon_{i}=\varepsilon^{\prime }-j\varepsilon^{\prime \prime }=\varepsilon^{\prime }-j\frac{\sigma }{\omega \varepsilon_{o}}$$

On the other hand, the EMI shielding can be expressed as how far a composite has transmitted the EMR, which can be explained by using the transmission coefficient (*T*). The *T* is depending on the transmission coefficient at the 0−*t* boundary (*T_1_* and *T_2_*), reflection coefficient at the 0−*t* boundary (*R_1_* and *R_2_*), where 0 is considered as 1 and *t* as 2, and complex propagation constant (*γ_m_*). The *μ*, *ε*, *j*, and *ω* affect the value of *γ_m_* of the composite (Equations (20) and (21)).

(20) $$T=\frac{T_{1}T_{2}e^{-\gamma_{m}D}}{1+R_{1}R_{2}e^{-2\gamma_{m}D}}$$

(21) $$\gamma_{m}=j\omega \sqrt{\varepsilon_{o}\mu_{o}(\varepsilon_{eff}^{\prime }-j\varepsilon_{eff}^{\prime \prime }}$$

**Scheme 1:**
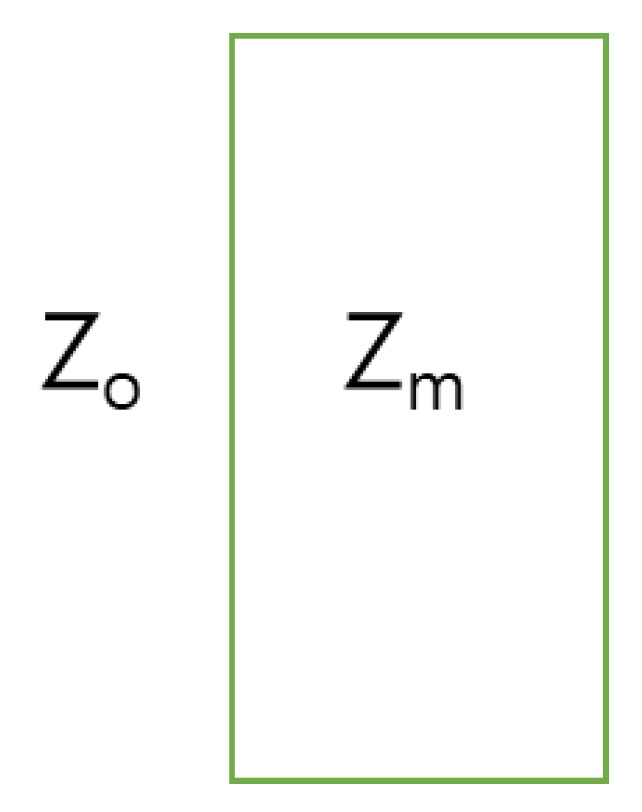
Indication of the *Z_o_* and *Z_m_* in a composite.

*Z_o_* and *Z_m_* determines the magnitude of *T*_1_ and *R*. Moreover, *Z_o_*, *μ_r_*, and *ε_eff_* have the impact on the value of *Z_m_* (Equations (22)–(26) and Scheme 1).

(22) $$T_{1}=\frac{2Z_{m}}{Z_{m}+Z_{o}}$$

(23) $$T_{2}=\frac{2Z_{o}}{Z_{m}+Z_{o}}$$

(24) $$R_{1}=\frac{Z_{m}-Z_{o}}{Z_{m}+Z_{o}}$$

(25) $$R_{2}=\frac{Z_{o}-Z_{m}}{Z_{m}+Z_{o}}$$

(26) $$Z_{m}=Z_{o}\sqrt{\frac{\mu_{r}}{\varepsilon_{eff}}}$$

Hence, the *SE* can be calculated in terms of *T* and the shielding efficiency of the materials can be calculated based on the *SE_T_* of the composite (Equations (27) and (28)) [9,38].

(27) SE=−20log(|T|)

(28) $$\mathrm{Shielding}\;\mathrm{efficiency}\;\left(\%\right)=100-\left(\frac{1}{10^{SE/10}}\right)\times 100$$

### 2.4. The MXene Composite Preparation Techniques

**Figure 8:**
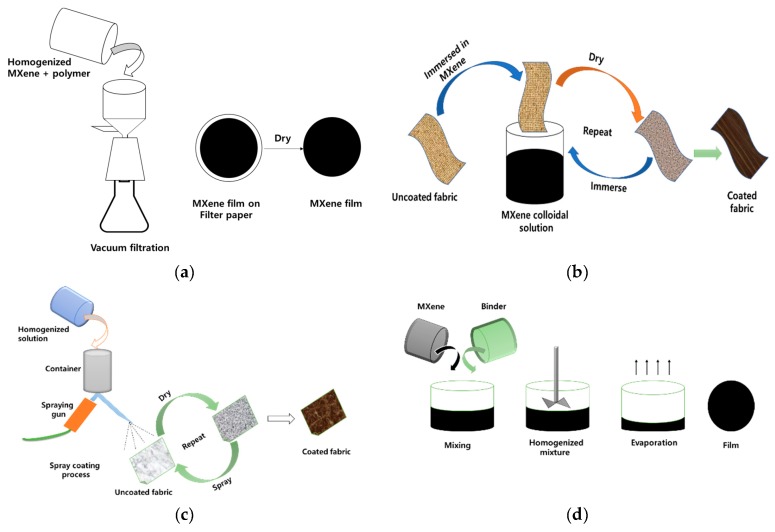

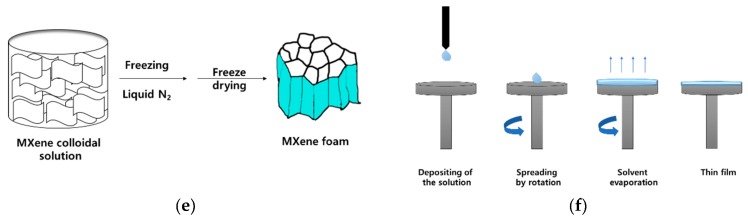
Schematic representation of: (**a**) vacuum assistant filtration (VAF), (**b**) dipped coating, (**c**) spray coating (**d**), solvent casting, (**e**) freeze drying, and (**f**) spin coating.

The composite of MXene and graphene are prepared by various processes such as vacuum assistant filtration (VAF) [38], dipped coating [39], spray coating [40], solvent casting techniques [41], freeze drying [42], and spin coating [43,44]. Practicing the processes varies based on the composition, purpose, and type of material used. The VAF technique is where a homogenized mixture is filtered through the filter paper and dried. The homogenization is carried out by sonication or a stirring process and various types of filters are being used such as paper, nylon, polypropylene, and Nuclepore track-etched polycarbonate (PC). Further, hybrid films are prepared by alternative filtering of the homogenized mixture (Figure 8a) [38]. In the dipped coating process, the matrix materials are immersed in the suitable solvent for a particular time period and dried, which is repeated several times based on its requirement (Figure 8b). Furthermore, the spray coating has a similar process (Figure 8c) [39]. In the spray coating process, spraying speed, time, solvents, pressure, and particle size of the materials in the solvent are controlled based on the quality of the products’ needs (Figure 8c) [40]. The solvent casting is done by evaporating solvent of the homogenized mixture in an air or vacuum oven and the evaporation time depends on the type of solvent used. Then, the film is separated from the casting plate (Figure 8d) [41]. In the freeze drying technique, the homogenized water mixture is frozen under liquid nitrogen, and then the composite dried at the same temperature. During this process various structures such as honeycomb, porous, and other types of foam composites are formed, which is dependent on the type of constitutional elements present in the homogenized mixture. The resultant product is mixed with proper binder to prepare EMI shielding composite and this new technique is widely used to create highly efficient EMI shielding material (Figure 8e) [42]. Spin coating is a general technique used to prepare various thin electronic devices for which high rpm, different substrate, and evaporation techniques are used (Figure 8f) [43,44].

### 2.5. EMI Shielding of MXene Composite and Synthesis

The EMI shielding of the MXene (MX) composite varies based on the components that are integrated with MXene and the structural features of the composites. The MXene/aodium alginate (SA) shows 92 dB of EMI shielding effect (EMI SE) with 45 μm of thickness (t) and 4600 S·cm^−1^ of electric conductivity (EC) which is the highest EMI shielding reported for MXene composition in 2016. Further, the EMI shielding of the MX/SA composites diminish with reduced thickness of the composite prepared by the vacuum assistance filtration (VAF) technique, giving rise to the nacre−like structure and display good EMI SE. The spray coating of pure MXene (10 mg·mL^−1^) on the PET surface displays EMI shielding of about 50 dB with 4 μm, which is almost similar to that of VAF MX/SA composition with 8-μm thickness. It is obvious that spraying on the polar surface increases the EMI SE while integration of the foreign element with MXene significantly reduces the EMI SE. Hence, the synthetic route influences the EMI shielding of the composite and the SA helps to arrange the MXene in a nacre array which significantly improves EMI SE of the shielding material (Figure 8 and Table 3) [45]. Hu et al. stated that the cellulose/MXene (M−filter) nanocomposite paper displays the 43 dB of EMI SE (seven cycle dipped coating) in both X and Ku band with 27.56 S·cm^−1^ of EC and thickness of 0.2 mm which is differ from the Coa et al. MX/cellulose composite performance. The MXene/cellulose composite exhibited EMI SE of 24 dB with SSE of 12 dB·cm^3·^g^−1^, 2647 dB.cm^2^·g^−1^, and 0.047 mm of thickness (Table 3) [39,46,47]. Hu et al. coated MXene with commercial filter paper (density of 0.49 g·cm^−3^) and then, utilized the polydimethylsiloxane (PDMS) for finishing purposes, denoted as PDMS−M−filter composite while Coa et al. used MXene/cellulose nanofiber (CNFs) derived from garlic husk and both practiced dipped coating and VAF, respectively. The nacre-inspired structure of MXene/CNFs, which is absent in dipped coating cellulose composite and present in VAF composite, is an inspired structural feature in the MXene/cellulose based composites. Further, the filler loading determines the EMI, EC, and tensile strength of the composite [39].

In addition, CNTs/MXene/cellulose nanofibrils composite paper prepared by facile alternating vacuum assisted filtration process give rise the 38.4 dB of EMI SE and corresponding EC and thickness are 25.066 S·cm^−1^ and 0.038 mm, respectively. The EMI SE of CNTs/MXene/cellulose is higher than that of MX/cellulose composite reported above. Thus, the introduction of the CNT enhances the EMI SE. A similar study reported by Raagulan et al. used MXene–carbon nanotube nanocomposites (MXCS) where carbon fabric is used instead of cellulose displayed 99.999% shielding ability. The EC of CNTs/MXene/cellulose nanofibrils composite is 2.12-times higher than that of MXCS. The difference of EMI shielding in both cases is due to the thickness, EC, and curved MXene multilayer structure [47,48]. Xin et al. described that intercalation of silver nanoparticle with MXene/cellulose composition enhanced EMI shielding and exhibited EMI SE of 50.7 dB with 46 μm thickness and 5.882 S·cm^−1^ of EC. In this case, the introduction of silver nanoparticle in cellulose and MXene composition formed a similar structure reported by Cao et al, and the silver ion causes self-reduction of MXene, which improved the EC, multilayer formation, dielectric constant, and conduction loss [49]. The fiber matrix helps to diffuse EMR and fillers and fibers attenuate the EMR. The study of Liu et al. showed that the aluminum ion reinforced MXene film exhibits the excellent EMI shielding of 80 dB with 2656 S·cm^−1^ of EC and 0.005 mm of thickness where aluminum ion plays a major role in EMI SE and tensile strength which is due to the cross link formation between MXene and aluminum ion. Thus, aluminum ion has a greater tendency to induce the EMI SE than sliver ion in the matrix of the composite [49,50]. Furthermore, conductivity of the aluminum reinforced MXene is lower than that of MX/SA composite which is due to the interlayer space caused by the aluminum ion and higher than that of MXene/poly(3,4−ethylenedioxythiophene) polystyrene sulfonate (MX/PEDOT: PSS), MXCS, and d−Ti_3_C_2_T_x_/CNFs reported (Table 3) [45,48,49,50].

The poly (vinyl alcohol)/MXene (PVA/MXene) multilayered composite reported by Jin et al. exhibits 44.4 dB (wt.%—19.5%) of EMI SE (SE_R_—8.3 dB) with 0.027 mm of thickness and decrease with decreasing MXene loading in the PVA matrix. MXene in a PVA matrix has a similar EC trend and diminishes with less loading of MXene filler (Table 3) [51,52,53]. Further, the Xu et al. study shows that the PVA/MXene foam considerably minimized the EMI SE and exhibits 28 dB of EMI SE with a lower SE_R_ (2 dB), which is due to the differences in EC and its porous nature. The SE_R_ above 3 dB is induced by charge flow in the matrix. It is obvious that the EMI SE of the PVA/MXene composites are contingent not only structural features, but also filler loading of the composite [53,54]. The honeycomb (HC) structure can be manufactured by using reduced graphene oxide (rGO)−MXene/epoxy composition for which the Al_2_O_3_ HC template is used. Initially, the rGO is adsorbed on the surface of the template and then it is dissolved by using hydrochloric acid. Consequently, the HC graphene oxide is immersed into the MXene/CTAB solution and freeze dried. The yielded HC−rGO/MXene is strengthened by epoxy polymer. The HC−rGO−MXene/epoxy composition displays EMI SE of 55 dB with 3.871 S·cm^−1^ of EC and 0.5 mm of thickness [55,56]. A similar study is performed by Bian et al. who prepared MXene aerogel without a template and its corresponding EMI SE of 75 dB and SE_R_ is about 1 dB [57]. The EC of HC−GO−MXene/epoxy composite is lower than that of MXene aerogel which is due to the interconnection between MXene and epoxy polymer comparatively lessened the EMI SE and EC. The epoxy polymer not only minimized the electron flow among MXene and graphene but also improved the tensile strength of the composite and flexibility [56,57]. Zhou et al. described that the MXene/calcium alginate aerogel ((MX/CA (t = 26 μm)) exhibits 54.43 dB of EMI SE which is higher than that of the MX/SA composite synthesized in the same condition (t = 14 μm) (Table 3) [58]. Although, the 8 μm MX/SA reported by Shahzad et al. exhibited 57 dB of EMI SE which is assumed as the quality of the exfoliated MXene synthesized and fabrication condition used (Table 3) [46,58]. Further, the thickness or inter space of the composite is increased by types of interacting ion or organic substances used which also affect the EMI SE parameters. The thickness of the MX/CA is higher than the MX/SA which is caused by calcium alginate and aerogel structure of MX/CA diminished the EC and increased the corresponding SSE/t of the composite (Table 3) [58]. 

The interposing of the rGO into MXene formed, MX/rGO aerogel shows 56.4 dB of EMI SE which is almost similar to the HC−MX−rGO/epoxy composite, thus, the HC structure is an effective feature with the lowest thickness and filling load [56,59]. Scientists recently focusses on the 3D aerogel which seems to be a more effective EMI absorbent than the planar composite. The 3D Ti_3_C_2_T_x_/SA (95%) hybrid aerogel coated by electrically conductive polydimethylsiloxane−coated (PDMS) displays EMI SE of 70.5 dB and corresponding EC is 22.11 S·cm^−1^. In addition, the higher amount of SA reduces the EMI SE and EC. Further, SE_A_ and SE_T_ are almost similar where SE_R_ seems null is an evidence that the aerogel structure greatly improved absorption and the direct interconnection of MXene is a crucial parameter for EMI SE and SE_A_ [42]. Further, introduction of carbon into the MXene framework (MXene/carbon foam (MCF)) showed less EMI SE of 8 dB and addition of epoxy polymer followed by annealing of MCF reached up to EMI SE of 46 dB with 1.84 S·cm^−1^ of EC (2 cm of thickness). In this study, the resorcinol−formaldehyde sol−gel mixture is used as a precursor for carbon form, created by an annealing process, nonetheless, Song et al.’s epoxy composite consisted of rGO as a carbon form gave rise to higher EMI SE (55 dB) [56,60]. Hence, the filler with good electric conductivity and proper geometry improved EMI SE [60]. Furthermore, Wang, et al. reported that the annealed MXene/Epoxy Nano composites (wt.% 15) with 41 dB of EMI SE, 2 cm of thickness and 1.05 S·cm^−1^. From these studies, the annealing of composite internally created a carbon form from polymer that significantly changed EC and EMI SE. The direct annealing of pure MXene reduce the EMI shielding and due to the curing ability epoxy in MXene/Epoxy composite matrix enhances EMI SE [42,60].

Mixing of silver nanowire with MXene with cellulose pressured-extrusion method blocked 99.99% of incoming EMR, which is lower than that of pure MXene, and silver nanowire improved the electron flow path between 1D and 2D filler (Table 3) [45,61]. The corresponding composite assembled like brick-and-mortar like arrangement with internal pores which provide reflection and scattering interfaces that improve EMI SE [61]. Further, the heat treated monolayer MXene with 4.14 × 10^−5^ mm of thickness shows EMI SE of 17.13 dB and corresponding SSE and SSE/t are 7.17 dB·cm^3^·g^−1^ and 1.73 × 106 dB·cm^2·^g^−1^, respectively. The corresponding composite (without heat process) with the same thickness displays EMI SE, SSE, and SSE/t of 13.56 dB, 5.67 dB·cm^3^·g^−1^, and 1.37 × 10^6^ dB·cm^3^·g^−1^, respectively. It is apparent that the annealing at 600 °C improve EMI SE and other parameters significantly [43]. Wan et al. produced MXene/PEDOT: PSS composite with 6 µm of thickness gave rise to 40.5 dB of EMI SE while the same composition reported by Liu et al. showed 42.1 dB of EMI SE with 11 µm of thickness from which the preparation method plays a major role in determining the EMI SE. The removal of PSS from the matrix increase EMI shielding with 6 µm of thickness which is the main reason for Wan et al.’s results [38,62,63]. The SE_A_ mostly depends on the dielectric properties of the EMI shielding composite. Han et al. alter the surface of the MXene by annealing at 800 °C under the inert environment and prepare the MXene/wax composite (1 mm) display show EMI SE of 76.1 dB with 67.3 dB of SE_A_ and corresponding composite exhibit −48.4 dB of minimum reflection coefficient which is due to the formation of the titanium oxide on the surface of the MXene [64,65].

### 2.6. EMI Shielding of Graphene Composite

**Figure 9:**
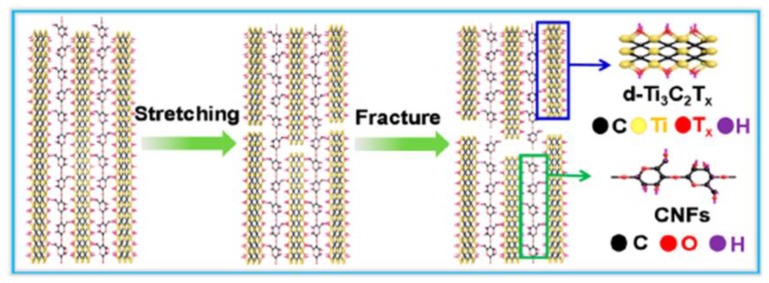
Interaction of the cellulose fiber with MXene and nacre−like structure formation [46] Copyright American Chemical Society, 2018.

Graphene is a 2D material and similar to MXene, whereas EMI shielding of GN depend on number of layers in GN and increasing layers enhance EMI SE of GN (EMI SE of single layer GN is 2.27 dB) [18]. The nacre-mimetic structure is an inspired structure for 2D materials such as MXene and GN which exert excellent EMI shielding (Figure 9). The graphene/PDMS polymer aerogel composite shows EMI SE of about 65 dB with the low GN loading (0.42 wt.%) and corresponding SSE and density are 100 dB·cm^3^·g^−1^, 0.0042 g·cm^−3^, respectively [8]. The physical or chemical process of the constitutional element of the composite determine the EMI shielding and other parameter of the composite. Further, the (thermally reduced graphene oxide−carbon nanotubes (TG−CN)−poly (methyl methacrylate) (PMMA) composite displays EMI SE of 30.4 dB while chemically reduce−GO−carbon nanotube/PMMA composite exhibits 1.68-times lesser EMI SE. For the reduction purpose, hydrazine and 900 °C were used and this process has the impact on structure of the foam, polarization loss, and multiple reflection of the composite. Additionally, chemical reduction process greatly reduces the EC of graphene−carbon nanotube composition whereas thermal process considerably improves free electron path in the composite [46,66,67,68]. Yang et al. handled another strategy to create the composite aerogel that is epoxy copper nanowires/thermally annealed graphene aerogel (6.0−1.2 wt.%) and corresponding EMI SE is 47 dB with EC of 1.208 S·cm^−1^, and 2 mm of thickness. Introduction of the copper significantly improved EMI SE and the EMI SE of the pure epoxy resin by 2 dB [69]. According to the Liang et al, report 32.5 dB of EMI SE was achieved for graphene/SiC−nanowires/poly(vinylidene fluoride) composites with a thickness of 1.2 mm and 0.015 S·cm^−1^ of EC. Thus, copper nanowire is the best choice compared to SiC−nanowire, though, SiC nanowire possesses good dielectric properties and enhances the absorption of the composite. Henceforth, the penetrating EMR is converted into heat energy in the 1D−2D network which is due to the ohmic loss greatly improved SE_A_ [1]. The addition of the inorganic component into the composite influenced the EMI SE, EC, magnetic property, and reflection loss. The cobalt-rich glass-coated microwires with the formula of Co_60_Fe_15_Si_10_B_15_ graphene/silicone rubber composite prepared blocked 98.4% of incident radiation and the corresponding filler loading is 0.059 wt.%. Furthermore, the composite consists of magnetic property due to the presence of the cobalt−ion containing microwire. The microwire (M) and graphene (G) arrange with the most prominent shielding structures, such as MMMGGG and MGMGMG. The microwire plays a dominant role in EMI SE, which is inferred that higher loading of M with proper geometrical array (MMMGGG) shows greater EMI SE than that of GN. This is due to the magnetic behavior of the M, polarization relaxation, and impedance matching. In addition, the dispersion array of the M and GN lower the EMI SE. According to the Xu et al., the multiple array of one type fillers followed another type of filler significantly enhanced the EMI SE [70].

The nickel foam can be created by a solution combustion (SC) technique and the resultant foam made up of Ni and NiO is new type of metal foam. By immersing Ni foam into the rGO solution (10 mgÅmL^−1^) for 2−3 h, the Ni-based EMI shielding composition can be fabricated. During the immersing period, the rGO nanoplates penetrate into the skeleton of Ni−foam which gives rise to a remarkable structure that enhances the EMI SE. The maximum reflection loss (RL) of Ni−GN foam is −53.11 dB and the corresponding thickness, porosity, dielectric loss range, complex permeability range, and density are 4.5 mm, 96.74%, 0.44–0.55, 1.05−0.97, and 38.54 mg·mL^−1^, respectively. The rGO majorly contributes dielectric loss to the Ni/NiO framework and Ni/rGO foam showed relatively higher μr than individual constitutional elements. Hence, nickel/rGO foam is a better choice than Cu nanowire/graphene aerogel [69,71]. Zhu et al. reported another type of composition that is Fe_3_O_4_/graphene coated Ni foam/poly dimethylsiloxane composite reached EMI SE of 32.4 dB with 2.5 S·cm^−1^ of EC and 1 mm of thickness. Introduction of the Fe_3_O_4_ and coating of the graphene on the Ni foam considerably affected EMI SE. The composite preparation method and type of binder used affects the shielding efficiency of the composite (Table 3) [67,69,71]. Li et al. made polyurethane based GN composite that is polyurethane/graphene (PUG20) composite with twenty percentage of weight ratio (20 wt.%) blocks 98.7% of incident radiation while epoxy encapsulated pyrolyzed PUG20 doubled the shielding ability (shielding power—99.99992%). The pyrolysis process not only improved GN content but also enhanced the porous nature, electrical conductivity, impedance mismatching, and SE_A_, and a higher filling load was attenuated at 99.99999% of the incident wave (Table 3) [4,43,45]. Graphene with the loading rate of 8.9 vol.% is dispersed in thermoplastic polyether block amide (Trade name—PEBAX) by heat process (175 °C) exhibits 30.7 dB of EMI SE and half volume loading of the graphene give rise to 16.6 dB of EMI SE.

In addition, the SE_A_ of GN/PEBAX is dominant factor like another composite reported above [72]. Thus, pyrolysis of the GN based polymer composite greatly improve EMI shielding and other parameters such as EC, dielectric property, porous structure, permeability, etc. The CNTs co−decorated porous carbon/graphene/PDMS foam generate 48 dB of shielding ability and corresponding SSE is 347.8 dB·g^−1^·cm^3^. The composite consists multilayer of components those design is better than that of the Zhu et al. study. The Ni foam is coated by CNT (GF) by the CVD method and Fe−Zn co−precipitated on hydrothermal method (FCC−GF) which is dispersed in the PDMS matrix [G11]. The Zhu et al. study just used an electrostatic assemble of nanomaterials on the porous structure that has less effect on EMI SE and CVD, or a hydro thermal process improved porous geometry and minimized the defect of fillers [67,73].

The many studies use PDMS as a binder to make GN-based EMI shielding materials. The 3D graphene/carbon nanotubes/polydimethylsiloxane composites (GC) was prepared by using freeze drying followed by annealing at a high temperature (2800 °C) and this is similar to the Song et al. study where they used the HC template to create the aerogel [58,74]. GC with 95.8% of porosity, 0.013 g·cm^−3^ of density, 2.4 mm of thickness, and 92.78 (vol.%) loading rate annealed at 1400 °C gave rise to 54.43 dB of highest EMI SE and the corresponding EC is 0.2012 S·cm^−1^. The higher annealing temperature (2800 °C) destroys EMI shielding of the composite, whereas the higher temperature repairs the defects by removing functional groups, reduced polarization loss, lessens the amorphous nature (higher amorphous nature at 1400 °C) and enhances the ohmic loss. Further, the introduction of the CNT prevents the agglomeration of GN and enhance the electric channels in the 3D network [58,74]. Cheng et al. prepared graphene−polyaniline (PANI)@polyimide composite with a ratio of 10:1 (GN: PANI) shows 21.3 dB (t − 0.04 mm) with 490.3 S·cm^−1^ of EC while GN/PANI, Ni decorated GN/PANI and Ag decorated GN/PANI (5 wt.%) show EMI SE of 24.85, 29.33, and 24.93 dB, respectively. Therefore, metal nanoparticle decoration with GN in PANI matrix improved EMI SE compared to the GN/PANI in polyimide matrix [68,75,76]. 

The electrical conductivity of the pure materials is high compared to the composite made from corresponding pure materials. Further, MXene based composition showed excellent EC compared to the GN. This is because of the functional group present in the MXene gave rise to a better arrangement in the polymer matrix which promoted a good electron flow path. Due to this, MXene is one of the most attractive materials in the modern electronic world. The SSE and SSE/t is depend on the density and thickness of the composite. Further, proper geometrical arrangement of the fillers is a crucial factor for better EMI shielding. 

## 3. Conclusions

In this review, we have highlighted recent progress in EMI shielding of MXene and graphene composite with different fabrication technique. The global requirement is a lightweight, flexible, cost−effective, and thinner EMI shielding material and EMI shielding over 20 dB, which is the basic requirement for the EMI shielding application in electronic devices. The graphene is synthesized by various methods in which radiative and chemical vapor deposition gives rise to good quality graphene and utilization of other methods is dependent on the types of applications. MXene synthesis also adapts various techniques and the lithium fluoride/hydrochloric acid etching is the most desirable etching method. The 2D MXene and graphene play a major role in creating different structures, especially aerogel. The freezing and freeze-drying process is used to prepare different geometric composite with excellent EMI shielding is a new approach. Further, the pyrolysis and hydrothermal process are important techniques used to achieve higher EMI shielding of the aerogel hybrid structure. The pyrolysis process of MXene created with surface titanium oxide, induces the EMI shielding while annealing of the MXene/polymer composite forms an internal conductive carbon network is another approach for excellent EMI shielding. The high temperature pyrolysis process of MXene or graphene polymer composite destroyed the EMI shielding function. The EMI shielding of graphene can be improved further by doping, decoration, oxidation, reduction, encapsulation, type of polymer, and processing technique. Thus, the proper geometrical arrangement of the fillers is a crucial factor for better EMI shielding and can be tuned by changing thickness, porosity, dielectric loss range, and complex permeability. The addition of inorganic nano composition like nanowire, ions, and nanoparticle further enhances EMI shielding and are being used to make the most powerful EMI shielding composites. The polymer/filler composites are explored to fulfil recent demand in EMI shielding in electronic devices. We hope, we have provided the fundamental understanding of MXene, graphene, and its corresponding composites to achieve a modern electronic goal in the near future. 

## Figures and Tables

**Table 1:** Categorization of the nanomaterial with examples and properties.

Dimension	Examples	Properties	Reference
OD	CdSe/V_2_O_5_ QDs, CdS/CdSe QDs, ZnO QDs, C−QDs, GN QDs, CoFe QDs, ZnCo QDs, metal hybrid QDs, SiO_2_ QDs, Au QDs	Improve microwave absorption, and magnetic properties, limited use because of narrow absorption bandwidth, high−density and perishable, large specific surface area, less thickness, can be mixed with other materials,	[10,11,12,13,14,15]
1D	single wall carbon nanotube (CNT) (SWCNT), multiwall CNT (MWCNT), decorated CNT (dCNT), nanowires (Ag, Cu, Si−C)	Carbonaceous materials show excellent chemical−physio−chemical stability, light weight, good complex permittivity, lack interfacial adhesion, deficiency of magnetic properties, poor dispersion, high production costs, and have impurities. Pure carbon materials show poor EMI attenuation and metal nanowire especially Ag greatly improve EMI shielding.	[10,11,12,13]
2D	2D−MXene, graphene (GN), doped GN, reduced GN, graphene oxide (GNO), nanoplates, hexagonal boron nitride (h−BN), layered double hydroxides (LDHs), transition metal dichalcogenides (TMDS), metal–organic Frameworks (MOF), layered metal Oxides (LMOs), covalent organic frameworks (COFs), metals, black phosphorus (BP) and silicene	MXene, GN and MoS_2_ are commonly used for EMI shielding, structure can be easily modified, MXene is excellent EMI shielder, nanoparticle decorated GN based composites show excellent EMI SE and functionalization of GN improve dispersivity, and di−electric properties, Pyrolyzed− MOFs/nanomaterials is used to design porous−magnetic high−efficient EMW absorption material, due to the synergy effect between magnetic loss and dielectric loss	[16,17,18]
3D	MAX phase, expanded graphite, graphite, metal plates, the 3D structure designed by using 0D, 1D, 2D nano structure and polymers	3D materials show less shielding ability, act as precursor to synthesis other nanomaterials, 3D structure made by mixture of nanomaterials show excellent EMI shielding properties.	[10,11,12,13,14,15,16,17,18]

**Table 2:** Comparison of different synthetic process of graphene.

Methods	Synthetic Method		Properties	Reference
Bottom−Up	Epitaxial growth of GN on SiC		SiC precursor, lacks homogeneity and quality, expensive due to energy consumption, have environmental concern because of tetraflu− oroethylene (C_2_F_4_)	[23,24,25,26,27]
	Dry ice method		Produced by complete burning of Mg ribbon inside the dry ice bowl.	
	Chemical Vapor deposition (CVD)		Is type of deposition process, gas phase precursors (CH_4_, C_2_H_4_, C_2_H_2_, and C_6_H_14_) are used, elevated temperature (450−1000 °C), metallic catalyst (Cu, Ni), low defective GN, and excellent electrical and optical property.	
	Template route		Good quality and well−defined structure, can get high yield by using pyrrole under mild condition, and less desire method due to the damage during purification.	
	Total Organic synthesis		Synthesis from polycyclic aromatic hydrocarbons, high quality GN with high yield, and limited size.	
	Substrate−free gas phase synthesis		New method, gas phase precursor (isopropyl alcohol and dimethyl ether and ethanol), clean and high quality GN.	
Top−Down	Arch discharge		Conventional method, used to synthesis fullerene, CNT and GN, high temperature plasma reaction (3727–5727 °C) in inert and air condition, and affordable cost.	[23,24,25,26,27]
	Liquid phase exfoliation		Common synthetic method, exfoliation occurs in aqueous and no−aqueous medium	
	Graphite intercalation		Intercalation of chemical species into graphite interlayer and improve electrical conductivity.	
	Radiation based methods		Short processing time, High quality, financially not viable, and radiation source are UV and laser.	
	Pyrolysis method		Solvothermal process, can be scaled up, good yield, and speed method.	
	Un−zipping of CNT− GN nanoribbon (GNR)		Cutting the cylindrical CNT by various methods (metal−catalyzed cutting, chemical unzipping, plasma etching, intercalation and exfoliation), low yield, and expensive precursors and chemicals.	
	Mechanical exfoliation		Use normal force (roll milling) and shear force (ball milling), high production cost, large processing time (24−48 h), low yield, and undesirable for large scale production.	
	Sonication		Ultrasonic energy, need large amount of energy, difficult to remove impurities, surfactants are used for sonication, and electrical conductivity.	
	Oxidative exfoliation and reduction	Thermal or hydrothermal reduction	Reduced to rGO, high temperature, greenhouse gas effect, and high operational cost.	
		Chemical reduction	Reduced to rGO and GN, many reducing agent are used (hydrazine (N2H4), zinc/hydrochloric acid, aluminum hydride, borohydrides, nitrogen−based reagents, sulfur−based reagents, sodium borohydride, microorganisms, and caffeic acid), lengthy synthesis time, additional chemical cost, environmental pollution, and toxic.	
		Electro−chemical reduction	Cost effective, less toxic, environmentally friendly, and rapid process.	
		Other reduction methods	photothermal, laser, microwave, photocatalytic, sonochemical, and plasma treatment	

**Table 3:** Electromagnetic interference (EMI) shielding comparison of the two−dimension (2D) MXene (MX−Ti_3_C_2_T_x_) and graphene (GN) composite.

No	Composite	Filler (wt.%)	t (mm)	SE (dB)	SEE (dB cm^3^·g^−1^)	SSE/t (dB·cm^2^·g^−1^)	Density (g·cm^−3^)	*σ* (S·cm^−1^)	Ref.
1	MX/SA	90	0.008	57	24.6	30,830	2.31	4600	[45]
2	MX/SA	bulk	0.011	68	28.4	25,863	2.39	−	[45]
3	d−Ti_3_C_2_T_x_/CNFs	90	0.047	24	12	2647	1.91	73.94	[46]
4	MXCS	bulk	0.386	50.5	324.15	8397.78	0.153	11.8	[48]
5	MX/PEDOT:PSS	87.5	0.011	42.1	2144.76	19,497.8	0.0196	340.5	[38]
6	MX/PEDOT:PSS	bulk	0.007	42.5	1577.08	22,529.7	0.0269	1000	[38]
7	MX/aramid nanofiber	90	0.015	32.84	20.05	13,366.67	1.638	628.272	[51]
8	MX/aramid nanofiber	80	0.02	30	20.65	10,325	1.453	173.36	[51]
9	MX/aramid nanofiber	40	0.022	19.43	16.36	7436.36	1.188	24.826	[51]
10	MX/GN	bulk	3	50.7	11021	36,736.67	0.0046	1000	[52]
11	PVA/MX	19.5	0.027	44.4	25.23	9343	1.744	7.16	[53]
12	PVA/MX	13.9	0.025	37.1	22.08	8833	1.68	3.79	[53]
13	Ti2CTx/PVA	0.15 (Vol.%)	5	28	2586	5136	0.0109	−	[54]
14	CNF@MX	bulk	0.035	39.6	24.6	7029	1.16	1.43	[55]
14	MX aerogel	bulk	2	75	9904		0.01	22	[57]
15	MX/CA aerogel	90	0.026	54.43	40.32	17,586	1.35	338.32	[58]
16	MX film	bulk	0.013	46.2	16.62	13,195	2.78	1354.29	[58]
17	MX/SA film	90	0.014	43.9	17.56	14,830	2.50	795.51	[58]
18	MX/AgNW film/Nanocell ulose	86	0.017	42.74	28.49	16724	1.5	300	[61]
19.	MX foam	bulk	0.06	70	318	53,030	0.22	580	[65]
20	MXPATPA	bulk	0.62	45.18	33.26	236.45	1.217	1.241	[31]
21	TG−CN/PMMA foam	10	2.5	30.4	43.4	173.6	0.701	0.0292	[66]
22	RG−CN/PMMA foam	10	2.5	18.1	26.2	104.8	0.691	0.0015	[66]
23	GN−CN/PMMA foam	10	2.5	25.2	47.5	190	0.531	0.013	[66]
24	Fe_3_O_4_/GN/PDMS	bulk	1	32.4	249.23	2492.31	0.13	2.5	[67]
25	Gr−PANI10:1@PI	40	0.04	21.3	16.38	4096.2	1.299	490.3	[68]
